# Anthelmintic Treatment of Sheep and the Role of Parasites Refugia in a Local Context

**DOI:** 10.3390/ani13121960

**Published:** 2023-06-12

**Authors:** Johan Höglund, Katarina Gustafsson

**Affiliations:** 1 Department of Biomedical Sciences and Veterinary Public Health, Section for Parasitology, Swedish University of Agricultural Sciences, 750 07 Uppsala, Sweden; 2 Gård och Djurhälsan, 514 05 Länghem, Sweden; katarina.gustafsson@gardochdjurhalsan.se

**Keywords:** anthelmintic resistance, livestock, control strategy, dose-and-move

## Abstract

**Simple Summary:**

The control of gastrointestinal parasites in sheep is often based on the integrated use of anthelmintics and pasture management. As problems with resistance to anthelmintics become more common, it is important to consider the timing of treatment. This is because one way to delay selection for anthelmintic resistance is to take into account that there are susceptible parasites in refugia that are not selected for treatment. In Sweden, stabled ewes are sometimes treated before being let out to pasture with their lambs in the spring. It is thought that this may increase selection for resistance. However, unlike in many other parts of the world, in Sweden, treatment is usually motivated by large numbers of worm eggs detected in faecal samples. In this review, we discuss whether the Swedish model is a risky strategy. In doing so, we consider the experience from several studies conducted in Sweden and abroad. We conclude that the evidence is inconclusive. Moreover, there are gaps in our knowledge, not least regarding the genetic background of resistance formation and how this is influenced by refugia. Further fundamental work is therefore needed on this topic.

**Abstract:**

Gastrointestinal nematodes in grazing livestock are ubiquitous and can cause severe damage, leading to substantial losses in agricultural yields. It is undeniable that the integrated use of anthelmintics is often an essential component of successful intensive livestock management. However, anthelmintic resistance has been a major challenge for several decades, especially in pasture-based lamb production. Measures are therefore needed to reduce the risk and prevent further spread. In many countries with more extensive lamb production and pronounced resistance problems than in Sweden, the importance of keeping parasites in refugia is emphasised. To ensure that treatment is necessary, the Swedish model is based on deworming certain groups of ewes based on the parasitological results of a faecal examination and then releasing them with their lambs to safe pastures. This is intended to reduce the risk of infection, which ultimately reduces the number of subsequent treatments. Whether this preventive strategy in turn means an increased risk of resistance is debatable. In this review, we explain the importance of parasites in refugia and how they can help delay the development of resistance to anthelmintics. We also discuss how likely it is that our model contributes to an increase in resistance risk and whether there is reason to question whether it is a sustainable strategy in the long term.

## 1. Background

Parasite control strategies against gastrointestinal worm infections in grazing livestock are constantly evolving and being challenged. In many countries, the use of broad-spectrum anthelmintics in combination with pasture hygiene is still the predominant control strategy. However, extreme production losses and high mortality rates due to worm infections have been reported in recent years, especially in regions with intensive sheep farming in the southern hemisphere [1,2]. The situation seems predictable, as common anthelmintics have lost efficacy to varying degrees in several countries, in some cases after only a few years on the market [3]. The way these drugs are used must be considered in the local context if they are to remain effective in the long term. This is because it is unlikely that new substances will come onto the market in the foreseeable future.

To promote the sustainable use of anthelmintics, refugia-based approaches were introduced many years ago: (i) targeted treatments (TT), where the whole herd is treated based on knowledge of infection levels, and (ii) targeted selective treatments (TST), where individual animals are treated rather than the whole grazing group [4]. These approaches are thus evidence-based and simultaneously aim to prevent worm-related negative effects on production while maintaining the long-term efficacy of dewormers by maintaining a pool of parasites in refugia, i.e., parasites that are not exposed to the drugs [5,6,7].

In this review, we summarise what is known about how refugia-based strategies work in a local Swedish context. We also discuss whether the TT-based “dose and move” strategy that has been common in Swedish sheep flocks for decades should be considered risky for the emergence of resistance and highlight knowledge gaps. Our arguments are based partly on local experience and partly on examples from abroad.

## 2. Why Is Worm Control Important?

Grazing livestock are infected with strongyle nematodes to varying degrees worldwide, with some species being of greater importance than others. In Swedish sheep, 16 different strongyles have been found, of which the gastrointestinal worms *Haemonchus contortus* and *Teladorsaia circumcincta* are the most common species [8]. The bloodsucking worm *H. contortus* is of particular concern as it can cause death in both growing lambs and pregnant ewes if they are heavily infected [9]. However, it should also be borne in mind that even moderate mixed infections are associated with reduced animal welfare and subclinical production losses [10].

These parasitic worms have in common that they infect their hosts while grazing. The eggs laid by the adult worms are spread over the pasture with the faeces of the host and soon develop into infective larvae. The more adult worms that are present, the more eggs that get into the grass, increasing the risk of infection. Parasitism is therefore a challenge, especially in intensive production, as more animals per unit area bring a greater risk of contamination of the pasture with harmful levels of larvae [11]. A well-functioning control strategy is therefore necessary to avoid the welfare and health problems associated with the loss of productivity. When affordable anthelmintics with a broad spectrum of activity became available in the 1960s, many farms succeeded in controlling worm infections [12]. For decades, the advantage of the strategic use of these drugs was undeniable because, unlike many alternatives, their efficacy is well documented and the substances are also easy to apply. In recent decades, however, several examples have shown that many anthelmintics no longer work. The reason for this is the increasing problems with the development of resistance of parasites to all major classes [13]. At the international level, resistance to anthelmintics is classified as a threat to global food security [14].

## 3. What Causes Resistance?

Anthelmintic resistance (AR) is defined as the ability of a parasite to survive a normally effective standard dose of a deworming drug [15]. Parasites are often characterised by large populations and the mixing of their genetic material through recombination, resulting in massive genetic variation. At the same time, effective unidirectional selection takes place when worms survive treatment, and their genes are passed on to the next generation.

As a result, the genes associated with resistance proliferate in the parasite population and soon take over [16]. According to the research findings, the resistance mechanisms are more or less unique to each substance class. For example, it can result from mutations, insertions, or deletions in positions that code for the proteins to which the drug binds and exerts its effect. When nucleotides are replaced, omitted, or added, the protein structure changes and the antiparasitic effect is weakened [17]. Other reasons for resistance may be that the parasite receives a lower dose of the drug because the enzymes involved in the breakdown of the drug or the transporters that regulate the excretion of the drug are affected. Although the exact mechanisms are not equally well characterised for all anthelmintics, experiments have shown that AR can evolve within a few generations, at least when reinfection occurs with surviving worms exposed to gradually increasing concentrations [18]. Although this chain of events is likely to be slower at the farm level, the experiments demonstrate the principle of resistance development.

## 4. How Can the Risk of Anthelmintic Resistance Be Reduced?

The selection of AR is delayed for obvious reasons if the use of anthelmintics is avoided. However, it seems unrealistic to stop deworming completely, not least because there are few scientifically proven alternatives with similar efficacy. For example, there are no recombinant vaccines against worm infections in grazing animals, despite years of intensive research. This is partly due to technical difficulties in production, but probably also has biological reasons, as natural protective immunity against strongyles is acquired slowly and is never sterile [19]. Regardless, this means that the age group normally most affected by the parasites, namely first-season grazing lambs, are unlikely to be effectively protected by vaccination. In adult animals, immunity can also be disrupted during pregnancy, lactation, and by poor nutrition. Trials of alternative measures, such as sowing pasture plants with anthelmintic properties or using nematode-trapping fungi, have also proved ineffective in our latitudes and are at best a supplement [20,21]. Moreover, some results are inconsistent or even contradictory. It is therefore unlikely that alternative solutions will soon be available that are as effective as modern anthelmintics.

If the use of anthelmintics is justified according to the principles of TT and/or TST, the frequency of treatment can be effectively reduced, and this is arguably a sustainable strategy that delays AR. However, a prerequisite for success is the willingness and ability of farmers to sample animals, but also access to cheap and reliable diagnostic tools used in an effective way. However, it is not only the frequency of treatment that counts. For example, a UK study suggests that the risk of selection on AR is higher when the drug is underdosed [22]. To further minimise selection on AR, it is therefore necessary to: (i) calculate the dose correctly, preferably after weighing the animals; (ii) ensure that dosing equipment is calibrated; and (iii) ensure that the anthelmintic is administered and stored correctly. In addition, resistant worms can be introduced when live animals are purchased. This has been documented in Sweden [23] as well as in other European countries [24,25]. An important measure is therefore the use of functioning quarantine routines that ensure a high level of biosecurity and where parasite status is carefully checked before animals are allowed out to pasture [26]. To reduce the need to move sheep from one farm to another, farmers can also rely on artificial insemination wherever possible.

## 5. How Risky Is Dose and Move?

It is beyond the scope of this text to describe when and how animals should be examined or what different grazing strategies can be used. Instead, the timing of deworming, i.e., the question of when animals should be treated, is dealt with in a little more detail below. A topic that has been discussed internationally for some years is the question of the extent to which anthelmintic treatment carried out before grazing or in conjunction with a change of paddock, promotes selection for AR.

As mentioned earlier, when dewormed animals are moved to a parasite-free pasture, resistant worms that remain in the animals are spread and soon take control, and the ball is rolling. Proponents of refugia therefore argue that there should be a pool of susceptible worms in the pasture to which the animals are moved after treatment. This part of the worm population will dilute the resistant gene pool and thus delay the development of resistance. This line of thought was confirmed by simulations carried out as early as the mid-1990s [27]. In real life, the matter is probably more complicated. Careful consideration is also needed when balancing the risk of developing AR against the acceleration of parasite-related damage and subsequent production losses that can occur if animals are not treated. This is important because it increases the risk of reinfection, which in turn can increase the frequency of treatments and thus the selection of AR.

Experimental evidence that the dose-and-move strategy accelerates the selection of AR is inconclusive. For example, results from a field trial in Australia showed that deworming sheep in combination with moving them to a safe pasture with low numbers of infectious nematode larvae did not result in faster development of AR than when the same number of treatments were administered to sheep that remained in the same pasture [28]. On the other hand, grazing trials conducted in New Zealand concluded that dose-and-move increased risk. Two consecutive studies showed that resistance increased when dewormed animals were offered a pasture with a low parasite load. In contrast, it decreased when parasites were in refugia or when some animals were left untreated [29,30]. However, in these studies, the amount of AR was determined using an in vitro detection method, while no major differences in the efficacy of the drug were found when comparing the reduction in faecal egg counts. While this can be explained by differences in the sensitivity of the two detection methods, “normally” dewormed control groups were missing in these studies. Therefore, it is not possible to determine the extent to which the different strategies tested were selective for resistance compared to common practise in New Zealand, where lambs are sometimes dewormed six times per grazing season [31]. As mentioned above, several independent studies have shown that the frequent use of drugs is an important risk factor for resistance selection [32].

Furthermore, a risk assessment of interventions related to ivermectin resistance in *T. circumcincta* on more than 200 sheep farms in Western Australia, where refugia are sometimes extremely low in summer, found that the lack of refugia was not the most important and only reason for AR [33,34]. Although the same study could not test and confirm the hypothesis, it rather concluded that winter drenching was key to the development of AR and that sheep farmers who sought advice from veterinarians on worm control were about half as likely to develop ivermectin resistance as those who relied on other sources [33]. Furthermore, it should be noted that in the UK, for example, where refugia-based strategies have been enthusiastically advocated in recent decades (https://www.scops.org.uk/, accessed on 20 April 2023), the problem of resistance has nevertheless increased dramatically over this period [13]. From these observations, it can be concluded that the factors contributing to the development of AR are extremely complex and that the number of treatments with the same agent irrespective of refugia seems to be crucial, regardless of refugia.

## 6. In a Swedish Context

The sustainability of a refugia-based nematode control strategy involving the use of anthelmintics depends on several factors and the specific context in which it is implemented. Although this type of control strategy is intended to contribute to sustainable parasite control, there are several things to consider. Not least, it is important to promote an understanding of the basic principles of such control strategies, including the importance of compliance, the responsible use of medicines, and integrated approaches. Collaboration between researchers, veterinarians, farmers, and policymakers is crucial for raising awareness. In Sweden, nematode control on most commercial sheep farms is generally achieved by using anthelmintics at specific times. At the same time, however, it is recommended to combine treatments with complementary measures to achieve sustainable parasite control. These include, first and foremost, grazing strategies that optimise pasture management to improve animal nutrition and immunity and thus reduce the parasite burden. A major challenge in this context is to raise awareness of the benefits and risks of grazing freshly treated animals. It is therefore encouraging that the majority of larger sheep farmers in the country make use of the available advisory services [35].

Regardless of whether one assumes that dose-and-move promotes resistance, one must conclude that this strategy is usually effective and often provides a good degree of worm control. In both the short and long term, this results in a reduced need for deworming, especially when *H. contortus* is present, as otherwise, animals become infected quickly and at a high rate. This is shown by experience in Sweden, where it is common practise to deworm stabled ewes before release, and lambs at weaning if deemed necessary based on the results of faecal examinations [36]. This ensures that the accumulation of larval infections on the pasture is delayed, which in turn reduces the risk of extensive worm infection in growing lambs. According to a recent nationwide questionnaire survey, 65% of respondents on sheep farms regularly perform faecal examinations for nematodes [35]. In another study, we found that ewes in some flock groups had an average of thousands of mainly *H. contortus* eggs per gram of faeces before grazing [37]. If such animals are not treated, this means a poor start to the grazing period with a high risk of subsequent health problems in the lambs. In the case of worm species that overwinter on pastures, such as *T. circumcincta*, *Trichostrongylus* spp., and *Oesophagostomum* spp., it can be argued that some larvae are present in refugia anyway, namely in spring when the freshly treated animals are released after stabling or are moved to new, previously used pastures in summer. On the other hand, this strategy has been shown to pose a major risk when monepantel-treated lambs were moved to parasite-free (sterile) pastures that had not been grazed before [38]. Nevertheless, species that overwinter mainly as inhibited larvae, such as *H. contortus* [39], seem to be at greater risk of developing resistance than the other sheep nematodes whose larvae are better able to survive the winter on pasture. In other words, this suggests that keeping larvae in refugia, which can immediately infect a new host after the animals have been let out to pasture, minimises the development of AR. On the other hand, it could also be because worm species with lower reproductive potential and that produce fewer offspring have a lower risk of developing AR. In any case, *H. contortus* is known to be one of the most prolific sheep parasites, displacing the other trichostrongylids. However, this question needs further investigation under the local conditions prevailing in Sweden. Although it seems that *H. contortus* is the species that has mainly become resistant to the anthelmintics approved in Sweden, the other species are also harmful to varying degrees and are known to become resistant elsewhere. Since gastrointestinal nematodes usually occur as mixed infections, it is therefore important to study the problem without limiting it to *H. contortus*.

However, the decision not to treat the ewes before they are released in spring carries the risk of production losses and clinical disease, and thus the need for more frequent and intensive deworming of the entire flock. This is because *H. contortus* is one of the most pathogenic worms, with a high fecundity and eggs that rapidly develop into infectious larvae. However, even with the dose-and-move strategy, it is likely that a small number of unselected parasite eggs will be introduced, firstly in the form of eggs laid before treatment and therefore with limited contact with the anthelmintic, and secondly in the form of eggs in the faeces that are carried mechanically, e.g., via hooves. It has also been shown that in practise, individual animals often escape without having been dewormed when a larger group of animals is treated. This could explain why the resistance status for both ivermectin and benzimidazoles was good in the last systematically conducted survey in Sweden, although the targeted group treatment of ewes before grazing and/or lambs on pasture has been practised for a long time [40].

In the study, only two farms with benzimidazole-resistant *H. contortus* were found in about 40 randomly selected sheep flocks [40]. As a result, deworming with ivermectin was recommended in flocks infected with *H. contortus*. Since then, the use of ivermectin in sheep has increased, while recently, resistance has been found in more and more farms [37]. The reasons for this are not yet clear, but it is thought to be mainly due to resistant worms being imported into the country and then spreading further [23]. Nevertheless, Swedish sheep are usually dewormed only a few times a year, and often with a focus on different groups of animals on different occasions [35]. Perhaps this also helps to maintain refugia.

**Figure 1:**
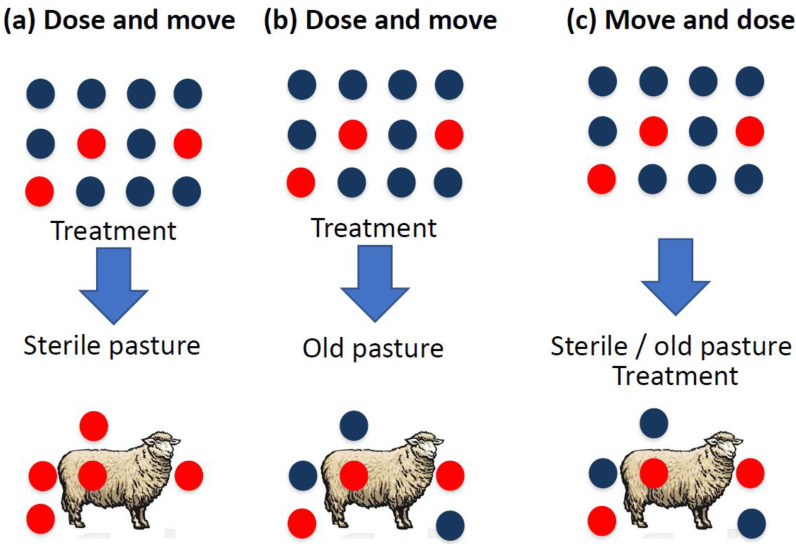
Schematic representation of the different outcomes of parasites in refugia shown in relation to the use of anthelmintics and the relocation of animals after treatment to different types of pasture (blue arrow). In (**a**,**b**), the animals are treated before the move, while in (**c**), they are treated a few days after the move. A sterile pasture is, for example, a newly established ley or a pasture that has never been used by sheep, while an old pasture was grazed by sheep the previous year or in the same season. Note that the figure does not claim to show the numerical ratio of resistant worms (red dots) to susceptible worms (blue dots).

Nevertheless, if one accepts that dose-and-move is a risky strategy with regard to the evolution of AR and therefore wants to work towards diluting the contribution of resistant genotypes to subsequent generations of worms in the farm, various measures can be taken. An important rule of thumb is to use highly effective dewormers (>99%) and leave a less random selection of the heaviest (resistant) animals untreated before moving them to a parasite-free pasture [41]. An alternative is to relocate some animals a few days before deworming, i.e., move-and-dose (Figure 1).

## 7. Conclusions

Although the arguments and underlying principles that refugia-based methods delay the development of drug-resistant parasitic worms are convincing, we must admit that knowledge of how refugia function under different farming conditions in different regions is limited. Several fundamental questions still need to be answered. For example, what is the role of conditions that influence the parasite population dynamics, such as the environment and production systems? What is the importance of the species composition of the parasites in combination with the choice of anthelmintic? What percentage of the susceptible population needs to be in refugia for the method to work, and how is this percentage estimated? Can we really predict the role of refugia without understanding the underlying genetic factors for each substance that can cause resistance? Therefore, finding out which genes are associated with resistance to different substances and how these traits are inherited is probably the most important piece of the puzzle.

All in all, a more precise understanding of how different anthelmintics can be applied in a safe way to delay resistance under field conditions is still a long way off. Undoubtedly, what is required for refugia to work and how transferable the strategy is between different parasite–host systems, in different climates and when using different drugs, are still largely unknown today. Finally, it is important to understand what proportion of animals can be left untreated to achieve a balance between effective parasite control and management of drug resistance. Therefore, further research on the underlying principles is needed to validate and optimise the use of refugia. Ultimately, we need to be clear that the goal of effective parasite control is ultimately to limit the number of larvae on pasture and thus reduce the risk of animals becoming infected with so many pathogenic parasites that this has negative consequences. The future challenges are undeniably numerous.

## Data Availability

The original data is available in the cited papers.
